# Supplementary material for: Föhn-induced melting over Larsen C modulated by atmospheric river shape, direction and landfall location
Source: Nat Commun. 2026 Apr 3;17:4788. doi: 10.1038/s41467-026-71359-2 (PMC13219627; doi:10.1038/s41467-026-71359-2)
Supplement: Supplementary file 1 — Supplementary Information [file 41467_2026_71359_MOESM1_ESM.pdf]

Supplementary information for: Föhn-Induced Melting over Larsen C Modulated by Atmospheric River Shape, Direction and Landfall Location

Xun Zou[1*], Penny M. Rowe[2], Irina V. Gorodetskaya[3], Andrew Orr[4], David H. Bromwich[5,6], Dan Lubin[1], Matthew A. Lazzara[7,8], Zhenhai Zhang[1], Brian Kawzenuk[1], Jonathan D. Wille[9], Jason M. Cordeira[1], Nicolaj Hansen[10], Jinxi Li[11], Pu Gan[11], F. Martin Ralph[1]

[1]Scripps Institution of Oceanography, University of California San Diego, La Jolla, CA, USA
[2]NorthWest Research Associates, Seattle, WA, USA
[3]CIIMAR | Interdisciplinary Centre of Marine and Environmental Research, University of Porto, Porto, Portugal
[4]British Antarctic Survey, Cambridge, UK
[5]Byrd Polar and Climate Research Center, The Ohio State University, Columbus, OH, USA,
[6]Atmospheric Sciences Program, Department of Geography, The Ohio State University, Columbus, OH, USA
[7]Antarctic Meteorological Research and Data Center, Space Science and Engineering Center, University of Wisconsin-Madison, Madison, WI, USA
[8]Department of Physical Sciences, School of Science, Technology, Engineering, and Mathematics, Madison Area Technical College, Madison, WI, USA
[9]Institute for Atmospheric and Climate Science, ETH Zurich, Zurich, Switzerland
[10]National Centre for Climate Research (NCKF), Danish Meteorological Institute, Copenhagen, Denmark
[11]Institute of Atmospheric Physics (IAP), Chinese Academy of Sciences (CAS), Beijing, China.

Corresponding Author: Xun Zou (x4zou@ucsd.edu)

This file includes:

1. Supplementary Figure S1-S8, and Table S1-S4.
2. Selection Criteria for AR Events and Föhn-Warming Periods, including Supplementary Figure S9-S11.

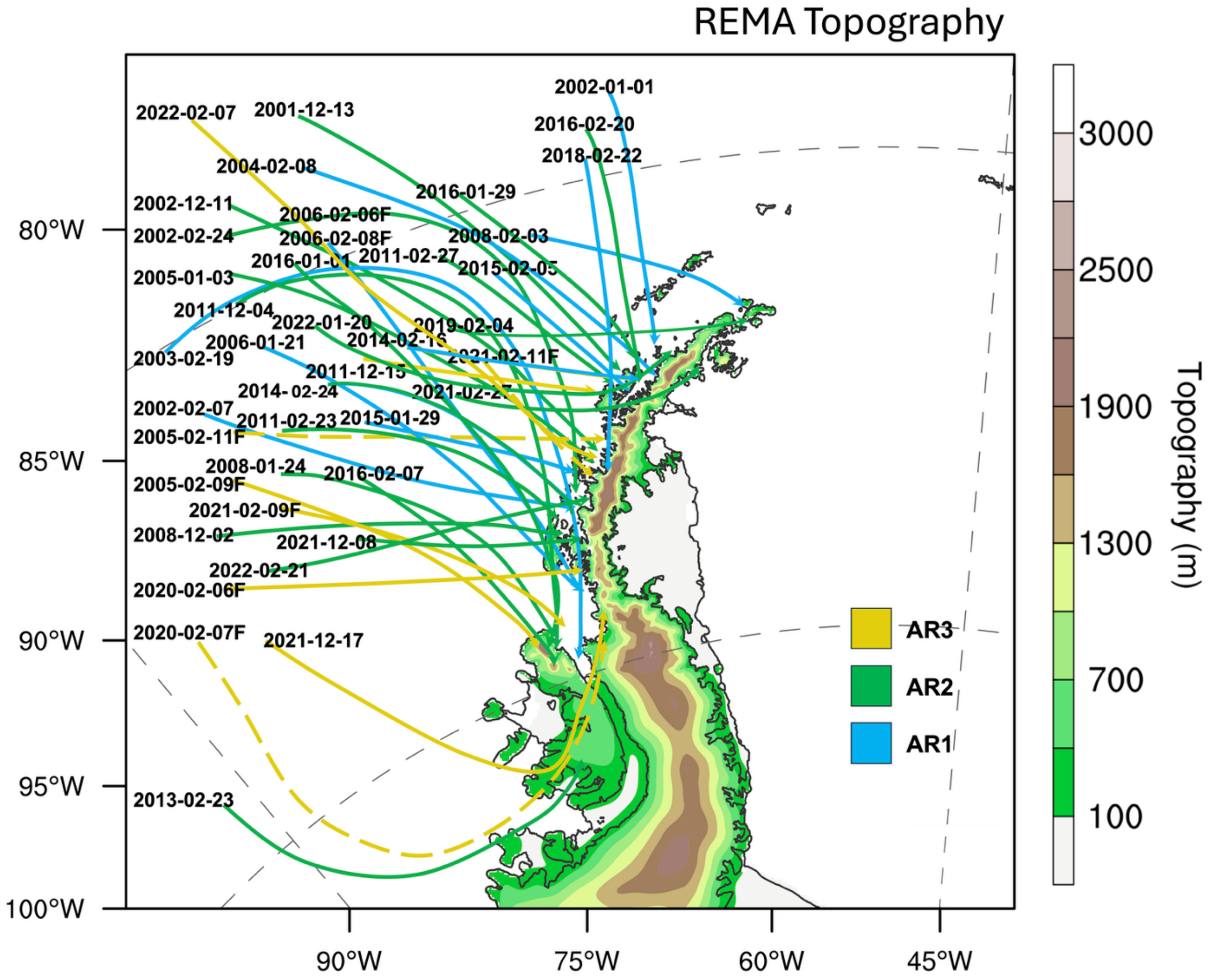

Supplementary Figure S1. Overview of selected atmospheric river (AR) events. Dominant AR shape for each AR-föhn event impacting the AP during austral summers from 2001 to 2022, as well as corresponding dates and ranking on the Enhanced AR Scale[1] (AR1 in blue, AR2 in green, AR3 in yellow). AR shapes of AR-family events are shown by dashed lines and a "F" after the date. If an AR-family event includes multiple shapes, the secondary shape is indicated by a dashed line. Also shown is the model topography (m) from D02 at 6 km, based on the REMA dataset. LCIS in (b) refers to the Larsen C Ice Shelf. The dominant AR shape is manually defined based on the Integrated Vapor Transport (IVT) field, following the AR axis at the peak of each event.

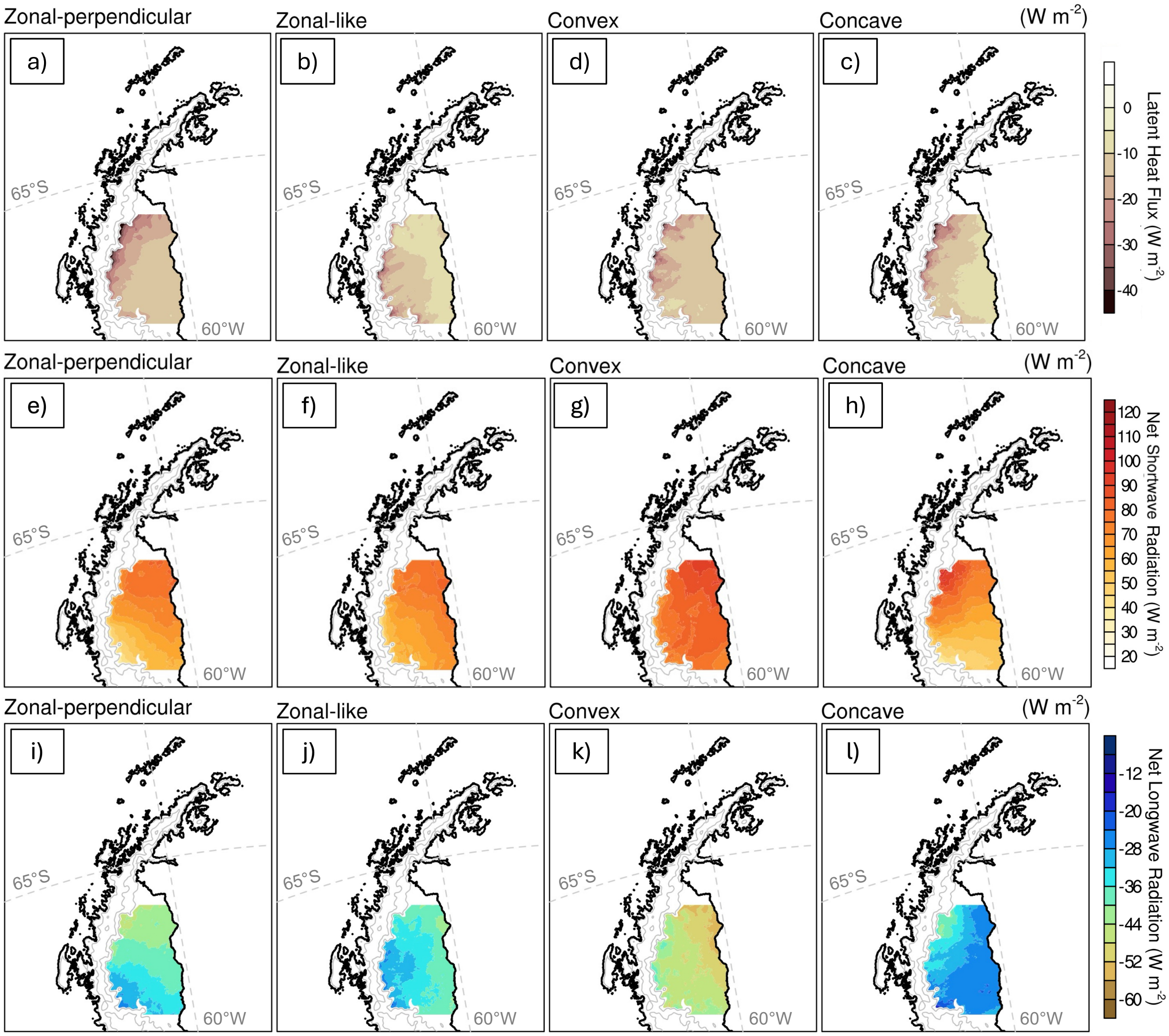

Supplementary Figure S2. Average values of surface energy balance for each atmospheric river (AR) shape. Based on the k-means clustering of Integrated Vapor Transport (IVT) shown in Fig. 2, composites of (a-d) hourly average latent heat flux (W m^{-2}), (e-h) hourly average net shortwave radiation (W m^{-2}) and (i-l) hourly average net longwave radiation (W m^{-2}) for zonal-perpendicular (a,e,i), zonal-like (b,f,j), convex (c,g,k) and concave (d,h,l) AR shapes.

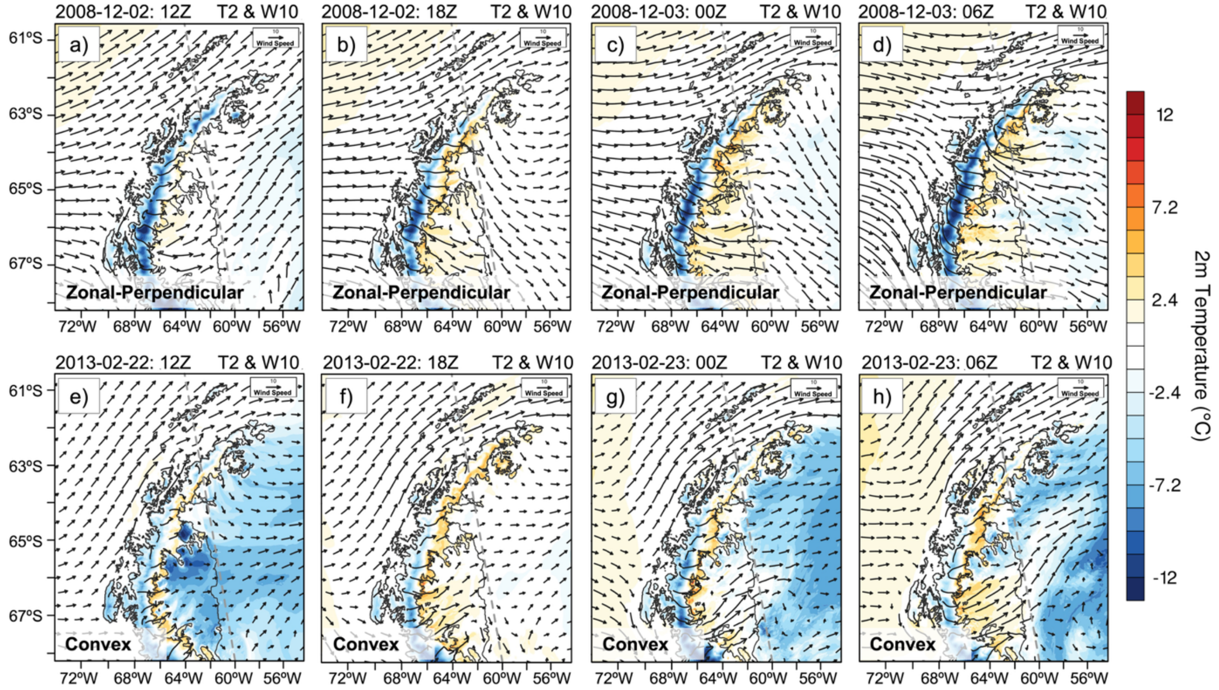

Supplementary Figure S3. Surface temperature and winds during two representative atmospheric river (AR) events. Evolution of 2 m temperature (T2; contour fill; °C) and 10 m wind (W10; vectors; m s^{-1}) of (a-d) zonal-perpendicular and (e-h) convex AR events, occurring in Dec 2008 and Feb 2013 respectively, and based on PWRF simulations. (a-d) shows results at 12Z 2 Dec, 18Z 2 Dec, 00Z 3 Dec, and 06Z 3 Dec, respectively. (e-h) shows results for 12Z 22 Feb, 18Z 22 Feb, 00Z 23 Feb, and 06Z 23 Feb, respectively. The length of the reference arrow at the upper right corner of each panel represents a wind speed of 10 m s^{-1}.

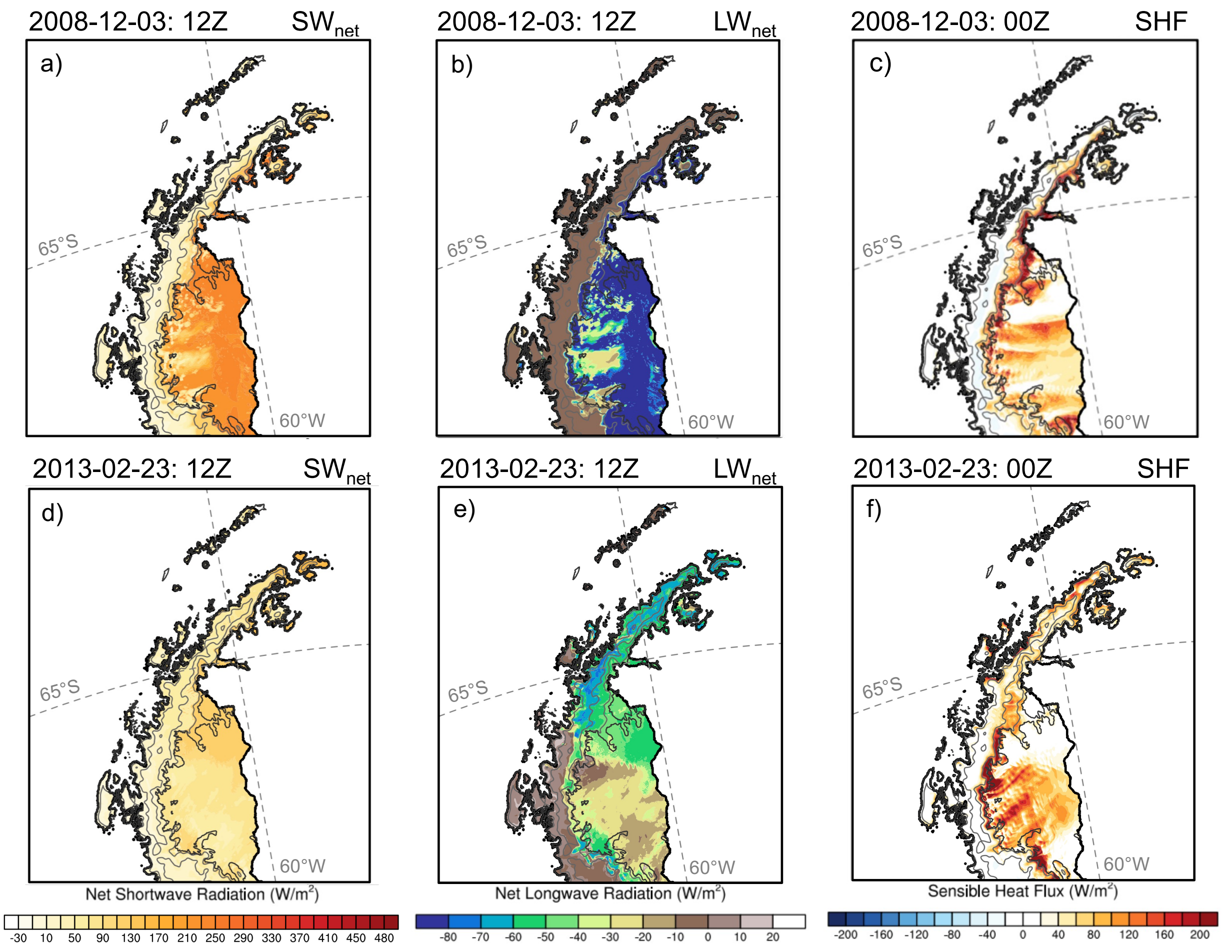

Supplementary Figure S4. Surface energy balance for two representative atmospheric river (AR) events. Surface energy balance components (W m^{-2}) of (a-c) zonal-perpendicular and (d-f) convex events, occurring in Dec 2008 and Feb 2013 respectively, and based on PWRF simulations. (a,d) net shortwave radiation (SW_{net}; W m^{-2}), (b,e) net longwave radiation (LW_{net}; W m^{-2}) at 12Z 03 Dec and 12Z 23 Feb, and (c,f) sensible heat flux (SHF; W m^{-2}) at 00Z 03 Dec and 00Z 23 Feb.

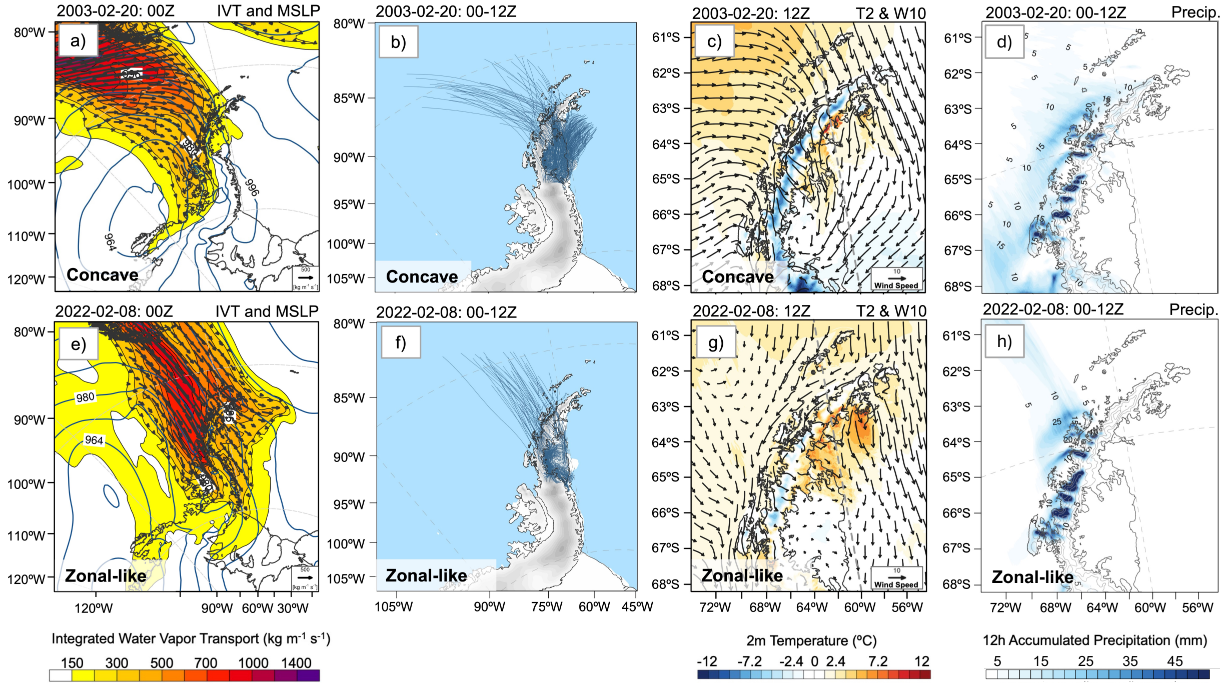

Supplementary Figure S5. Overview of two additional atmospheric river (AR) events. As Fig. 5 but showing (a-d) concave and (e-h) zonal-like AR events, occurring in Feb 2003 and Feb 2022, respectively, and based on PWRF simulations. (a,e) Integrated Vapor Transport (IVT; shading; kg m^{-1} s^{-1}) and Mean Sea Level Pressure (MSLP; contours; hPa) at 00Z on 20 Feb and 8 Feb, respectively. (b,f) 12-hour back trajectories ending over the LCIS from 00 to 12Z on 20 Feb and 8 Feb, respectively. (c,g) 2 m temperature (T2; °C) and 10 m wind fields (W10; m s^{-1}) at 12Z on 20 Feb and 8 Feb, respectively. (d,h) 12h accumulated precipitation (Precip; mm) from 00 to 12Z on 20 Feb and 8 Feb, respectively. The length of the reference arrow in (c,g) represents a wind speed of 10 m s^{-1}.

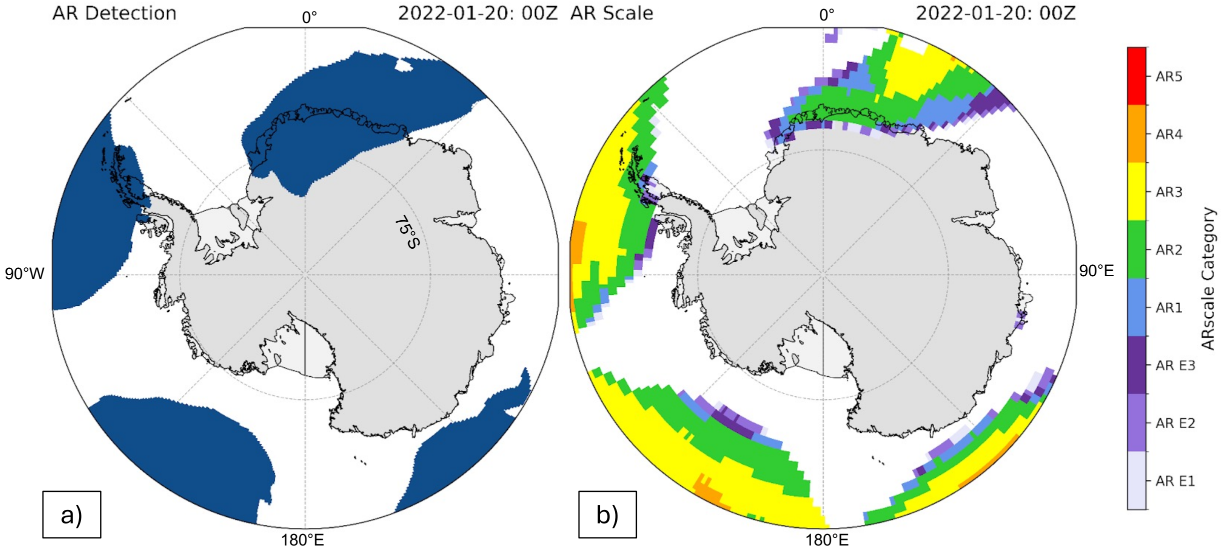

Supplementary Figure S6. Comparison of different atmospheric river (AR) detection algorithms. a) AR shape defined in Global Atmospheric Rivers Database (Version 4)[2] b) AR scale defined in Gridded Enhanced AR Scale[3] at 00Z on 20 Jan 2022. Comparisons for the remaining cases are available in the public database[4].

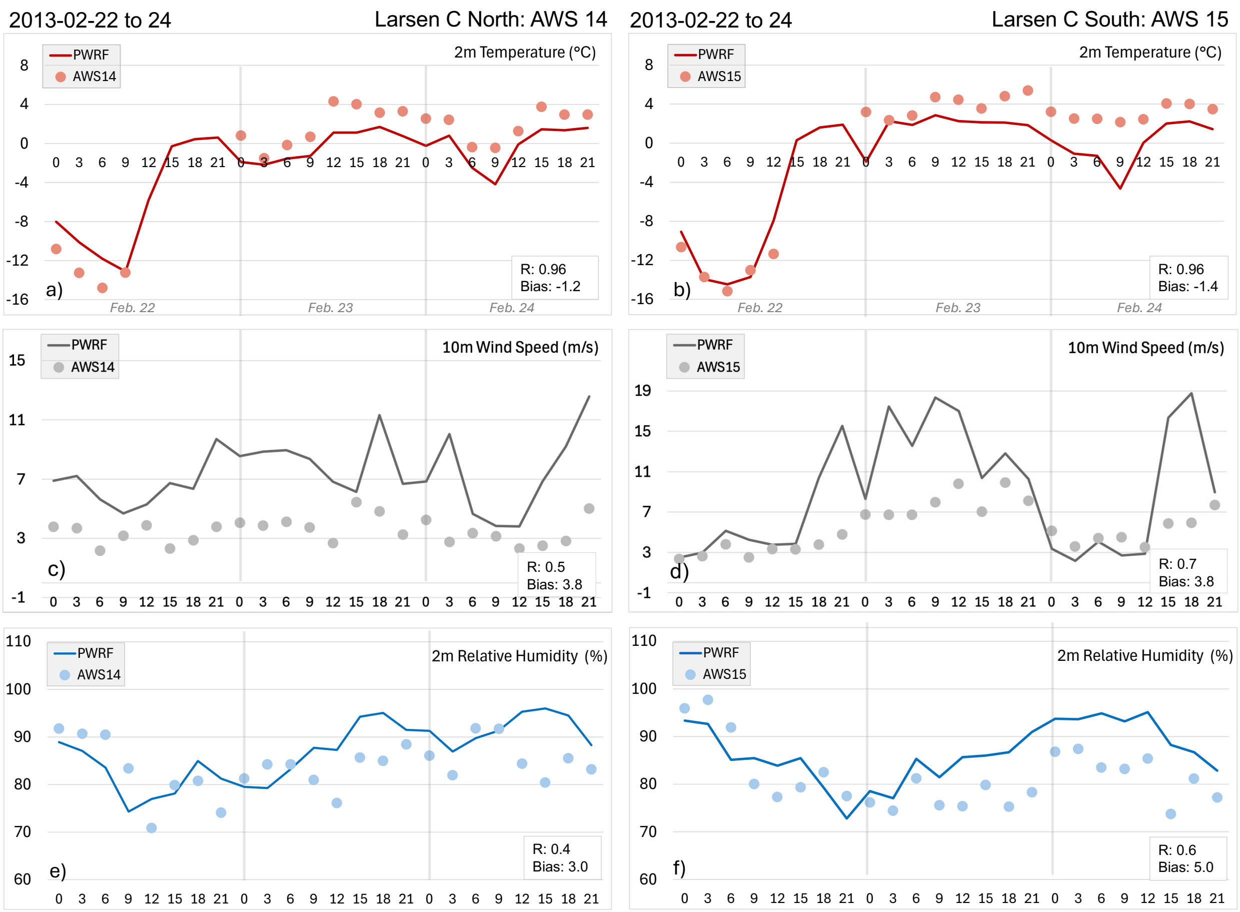

Supplementary Figure S7. Model evaluation of meteorological variables based on station observations. Comparison of PWRF simulations and Automatic Weather Stations (AWS) observations for a convex atmospheric river (AR) event occurring in Feb 2013. (a-b) 2 m temperature (T2; °C; red), (c-d) 10 m wind speed (W10; m s^{-1}; grey), and (e-f) 2 m relative humidity (RH2; %; blue) comparison at AWS15 and AWS14 over the Larsen C Ice Shelf from 00Z 22 to 24 Feb 2013. Locations of AWS14 and AWS15 are marked with red crosses in Fig. 1.

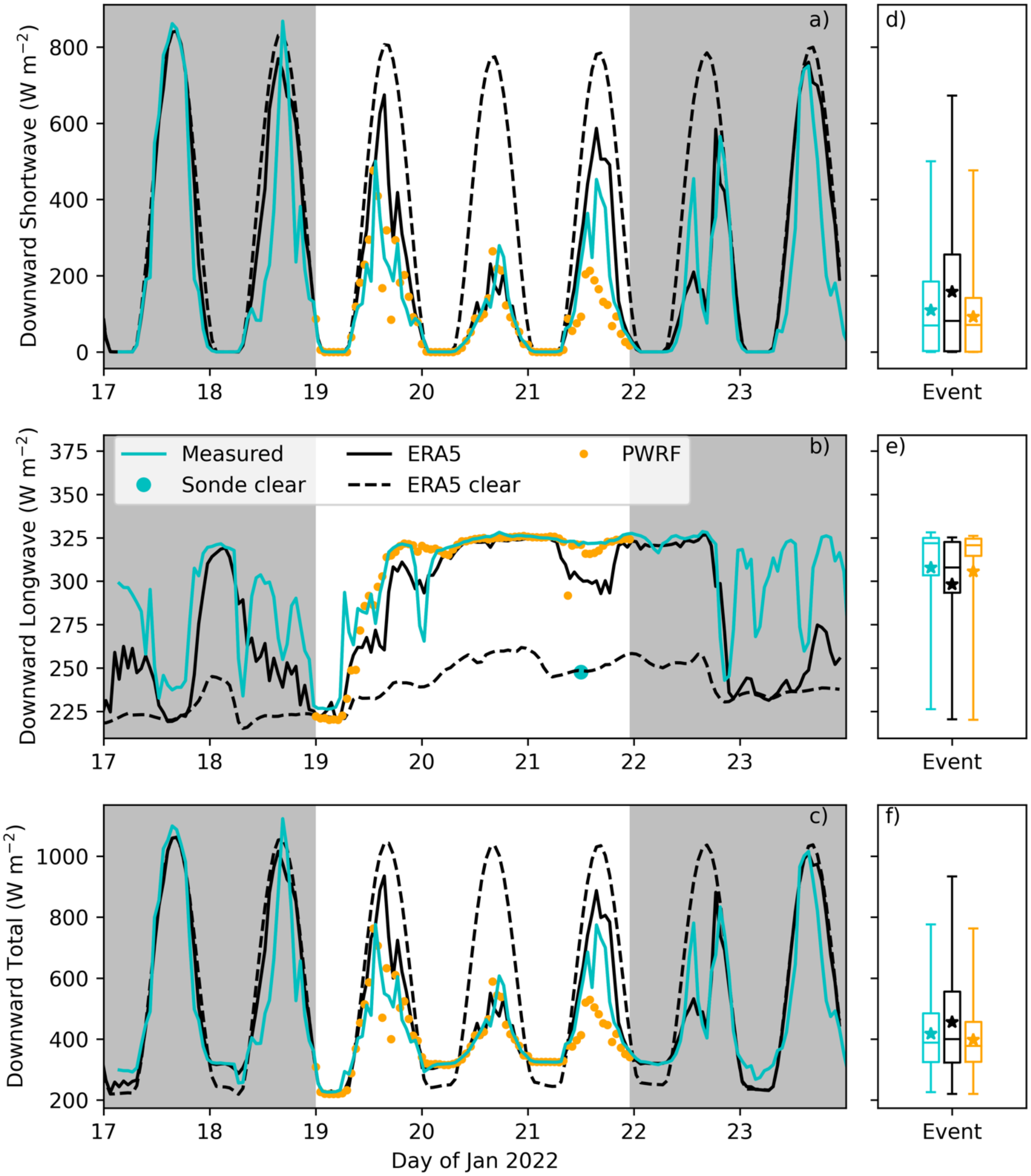

Supplementary Figure S8. Model evaluation for surface energy balance based on station observations. Time series of downward longwave and shortwave radiation at the surface at Escudero Station on King George Island during a combined AR-föhn event from 19 to 21 Jan 2022[5,6]. (a-c) observations (measured; hourly averaged; cyan), clear-sky simulations based on radiosonde measurements (Sonde clear), sky-view and clear-sky results from ERA5 (ERA5 and ERA5 clear; black) and sky-view results from PWRF simulations (orange). The AR duration is shown as the white span. (d-f) statistics for the duration of the AR event (white span in a-c), where the asterisk indicates the mean, the horizontal bar the median, the boxes the interquartile range, and the whiskers the full range. The legend in (b) corresponds to all panels.

Supplementary Table S1. Selected atmospheric river (AR) events. List of AR events impacting the AP during austral summers since December 2001[4], including their start and end dates, duration, AR scale, dominant AR shape, and the increase in hourly maximum 2 m temperature ($T2_{max}$) over the LCIS after removal of the diurnal cycle. Only warming periods lasting longer than 6 hours with $T2_{max}$ increases exceeding 3 °C over the LCIS are identified as föhn warming. "None" in the $T2_{max}$ column indicates that no föhn warming was detected. Asterisks (*) indicate AR-family events. The AR Scale denotes the maximum AR category detected on the upwind side of the LCIS, with a rating of 1 to 3 indicating moderate to strong AR events for Antarctica as suggested by previous research[1,7,8]. Bold text highlights the two cases involving zonal-perpendicular and convex AR shapes selected for detailed analysis in this paper.

No.	Start Date	End Date	Duration (days)	AR Scale (ERA5)	Dominant AR shape	$T2_{max}$ (°C)
1	2001-12-12	2001-12-15	4	AR2	Zonal-like	4.6
2	2002-01-01	2002-01-02	2	AR1	Fully meridional	None
3	2002-02-07	2002-02-08	2	AR1	Zonal-perpendicular	None
4	2002-02-24	2002-02-26	3	AR2	Concave	None
5	2002-12-11	2002-12-16	6	AR2	Zonal-like	4.7
6	2003-02-19	2003-02-21	3	AR1	Concave	4.9
7	2004-02-08	2004-02-10	3	AR1	Zonal-like	None
8	2005-01-03	2005-01-05	3	AR2	Zonal-like	5.8
9*	2005-02-08	2005-02-16	9	AR3	Zonal-perpendicular & Zonal-like	8.4 & 7.9
10	2006-01-21	2006-01-24	4	AR2	Zonal-like	7.5
11*	2006-02-06	2006-02-12	7	AR1	Zonal-like	3.8
12	2008-01-24	2008-01-27	4	AR2	Zonal-like	8.7
13	2008-02-23	2008-02-24	2	AR1	Zonal-like	None
**14**	**2008-12-01**	**2008-12-03**	**3**	**AR2**	**Zonal-perpendicular**	**4.4**
15	2011-02-23	2011-02-24	2	AR2	Concave	None
16	2011-02-26	2011-02-28	3	AR2	Zonal-like	None
17	2011-12-04	2011-12-06	3	AR2	Concave	None
18	2011-12-15	2011-12-17	3	AR3	Zonal-perpendicular	4.6
**19**	**2013-02-22**	**2013-02-24**	**3**	**AR2**	**Convex**	**8.4**
20	2014-02-15	2014-02-17	3	AR1	Zonal-perpendicular	None
21	2014-02-24	2014-02-26	3	AR2	Convex	4.9
22	2015-01-28	2015-01-29	2	AR1	Zonal-like	None
23	2015-02-05	2015-02-06	2	AR1	Zonal-like	None
24	2016-01-01	2016-01-04	4	AR2	Zonal-like	3.9
25	2016-01-29	2016-01-30	2	AR2	Zonal-like	None
26	2016-02-06	2016-02-08	3	AR2	Zonal-like	5.4
27	2016-02-20	2016-02-22	3	AR2	Fully meridional	None
28	2018-02-21	2018-02-23	3	AR1	Fully meridional	3.6
29	2019-02-04	2019-02-05	2	AR2	Zonal-perpendicular	None
30*	2020-02-04	2020-02-10	7	AR3	Zonal-perpendicular & Concave	12.7 & 5.3

31*	2021-02-08	2021-02-14	7	AR3	Zonal-perpendicular & Zonal-like	8.0
32	2021-02-27	2021-03-01	3	AR2	Zonal-perpendicular	5.2
33	2021-12-08	2021-12-10	3	AR2	Zonal-perpendicular	4.4
34	2021-12-17	2021-12-19	3	AR3	Concave	5.0
35	2022-01-19	2022-01-21	3	AR2	Zonal-like	3.7
36	2022-02-01	2022-02-03	3	AR2	Excluded	N/A
37	2022-02-07	2022-02-09	3	AR3	Zonal-like	3.9
38	2022-02-20	2022-02-24	5	AR2	Zonal-perpendicular	5.6 & 7.1

Supplementary Table S2. Summary of all atmospheric river (AR) shapes affecting the Larsen C Ice Shelf.

AR Shapes	Circulation	Impact Area	Representative Case
Zonal-like	High-low coupled system	Entire Larsen C, primarily north	2022-02-06 to 09
Zonal-perpendicular	High-low coupled system	Entire Larsen C	2008-12-01 to 03
Concave	Low pressure system	Northern Larsen C	2003-02-19 to 21
Convex	High pressure system	Entire Larsen C, primarily south	2013-02-22 to 24

Supplementary Table S3. PWRF model settings.

	PWRF V4.3.3
Input Data	ECMWF Reanalysis Data (ERA5)
Horizontal Resolution	30 km / 6 km / 1.2km
Vertical levels	71 levels (lowest level 4m above the surface)
Temporal Resolution	hourly
Spin-up	24 h
Microphysics	P3
PBL Scheme	MYNN
Shortwave and Longwave	Both RRTMG
Land Surface Options	Noah MP
Surface Layer Options	MYNN
Surface Albedo	MODIS observed surface albedo (MCD43C3)
High-Res Topo	Reference Elevation Model of Antarctica (REMA)1km topo
Nudging	Every 6 hours; nudging to u, v wind, temperature and water vapor from ERA5 fields (preprocessed and interpolated after the metgrid step)[9,10] for model level 40 (~400 hPa) and above.

Supplementary Table S4. Available stations for PWRF model evaluation from 2001 to 2015. Stations with observations during AR-föhn event periods defined in Supplementary Table S1 are included. Evaluation uses six-hourly output from domain D02.

Stations	Latitude (°)	Longitude (°)	T2 (R)	T2 Bias (°C)	T2 RMSE (°C)
Spring Point	-64.29	-61.05	0.67	-1.77	2.29
AWS17	-65.93	-61.85	0.63	0.88	2.49

Selection Criteria and Analysis Method for AR Events and Föhn-Warming Periods

1. AR Event Selection.

To select all available and qualified AR events, this study followed the steps below:

- Gridded Enhanced AR Scale calculation: We computed the area-averaged Enhanced AR Scale within the red dashed box (Fig. S9a) using the ERA5-based gridded dataset[3]. Only events with an average scale of AR1 or greater were retained.
- Landfall screening: A zoomed-in view over the Antarctic Peninsula (AP; Fig. S9b) was used to manually exclude events that did not directly impact the Larsen C Ice Shelf (LCIS; e.g., Integrated Vapor Transport - IVT > 250 kg m^{-1} s^{-1} confined to the Trinity Peninsula), cases in which the upwind side of the LCIS experienced AR intensity below AR1 at landfall, or events persisting for less than 24 h.
- AR scale assignment: The same zoomed-in plots were used to confirm and assign the AR scale for each retained event, defined by the highest AR scale detected on the upwind side of the LCIS. For example, the event occurring on 7-9 Feb 2022 is classified as an AR3, consistent with classifications reported in previous studies[7].
- 6-hourly IVT diagnostics: Six-hourly IVT fields from PWRF simulations were examined for each event (e.g., Fig. S10 for a three-day AR event in February 2022) to further confirm AR occurrence and to identify the dominant AR shape (Fig. S10e). Of the 38 events listed in Table S1, 37 making landfall on the upwind (western) AP were retained for further analysis (3,120 hourly time steps)[4].
- Cross-validation: Our selection results were cross-validated against an independent global AR detection dataset to ensure robustness[2] (e.g., Fig. S6), with all remaining events archived[4].

This relatively strict selection ensures the inclusion of all impactful austral-summer AR events over the LCIS while minimizing uncertainties associated with AR detection methods.

2. Föhn-warming Periods Selection

To identify all available föhn-warming periods based on changes in 2m temperature (T2) and 10m wind (W10) over the LCIS, this study followed the steps below:

- Temperature-based screening: For each event, we calculated the hourly maximum T2 ($T2_{max}$) over the LCIS (excluding the Scar Inlet) based on PWRF simulations, removed the diurnal cycle using all available time steps (n = 3,120), and identified candidate AR-föhn periods where the $T2_{max}$ increased by more than 3 °C and the warming continued for longer than 6 hours (e.g., Fig. S11a). The start of warming is defined as the onset of the $T2_{max}$ increase, and the end as when $T2_{max}$ decreases by more than 2 °C from its peak.
- Temperature and wind speed consistency check: These candidate periods were then cross-checked using 6-hourly T2 and W10 fields from PWRF D03 simulations to confirm the co-occurrence of surface warming and enhanced wind speeds over the LCIS (e.g., Fig. S11b). By the end of this step, 1,165 hours of simulations were selected.

3. K-means Clustering Analysis

K-means clustering analysis uses the Hartigan and Wong (AS-136) algorithm[11], which partitions multidimensional data points into K clusters by minimizing within-cluster variance. It was applied separately to 500-hPa geopotential height and IVT for all 1,165 selected hours of the PWRF D02 simulations and for additional pre-2001 events from the ERA5 reanalysis dataset (not shown). The number of clusters (k = 4) was determined based on manual inspection of all 6-

hourly IVT fields, which indicated that four distinct AR shapes occur during AR-induced föhn-warming periods.

Surface variables over the LCIS during AR-induced föhn-warming periods are analyzed based on IVT-defined clusters:

- T2: The diurnal cycle is first removed, after which the hourly rate of change of T2 is calculated. Hourly mean values over the LCIS are then computed for each cluster.
- Shortwave and longwave radiation (SW/LW); sensible and latent heat fluxes (SHF/LHF): Hourly mean values over the LCIS are calculated directly for each cluster.

4. Selection of Representative AR Shape.

Zonal-like AR shapes have been extensively examined in previous studies[7,12], concave ARs primarily affect the northern LCIS, and fully meridional ARs generally produce weaker impacts compared to other AR types. We therefore do not focus on these three AR shapes. In contrast, despite relatively weaker IVT magnitudes, both convex and zonal-perpendicular AR shapes generate moderate to strong warming over the LCIS. In particular, zonal-perpendicular ARs are theoretically more likely to trigger strong föhn effect due to their favorable airflow orientation relative to the AP topography. Therefore, we focus on these two AR shapes for further comparison.

For the convex AR shape, we selected the 2013 event, which ranks as the third-strongest föhn-warming case (the strongest convex case) over the LCIS among the 38 selected events (Table S1) and becomes the second when AR-family cases are excluded. We then selected a zonal-perpendicular event with a comparable AR intensity (AR2 lasting 3 days) and a similar landfall location, enabling a fair comparison that isolates the impact of AR shape (e.g., straight or curved).

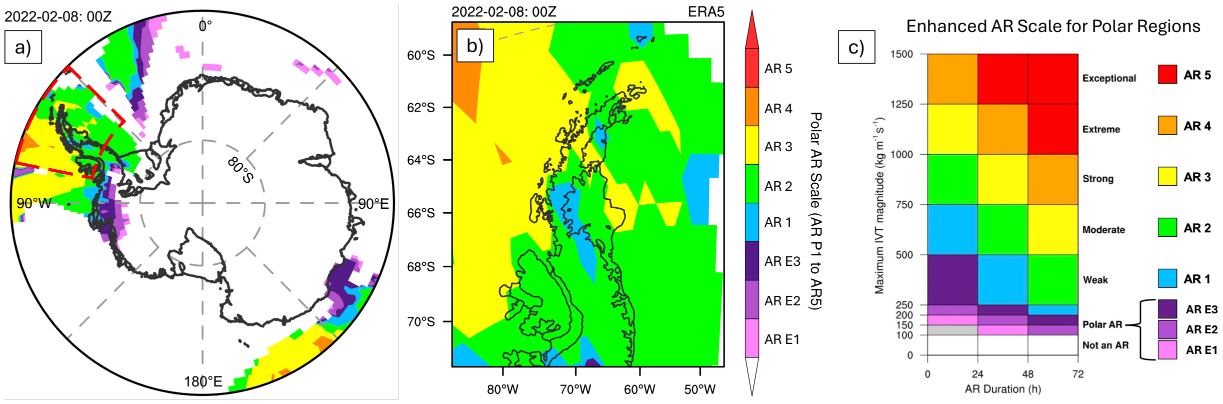

Supplementary Figure S9. Gridded Enhanced Atmospheric River (AR) Scale used for event selection. (a) Example of the gridded Enhanced AR Scale over Antarctica at 00Z on 08 Feb 2022 during an AR3 event[7,12]. (b) Zoomed-in view over the same time step, shown for cross-validation purposes. (c) Definition of the Enhanced AR Scale developed based on AR duration and the maximum integrated water vapor transport during the event. The red dashed box in (a) indicates the region used to compute the area-averaged gridded Enhanced AR Scale. This gridded dataset is derived from the ERA5 reanalysis.

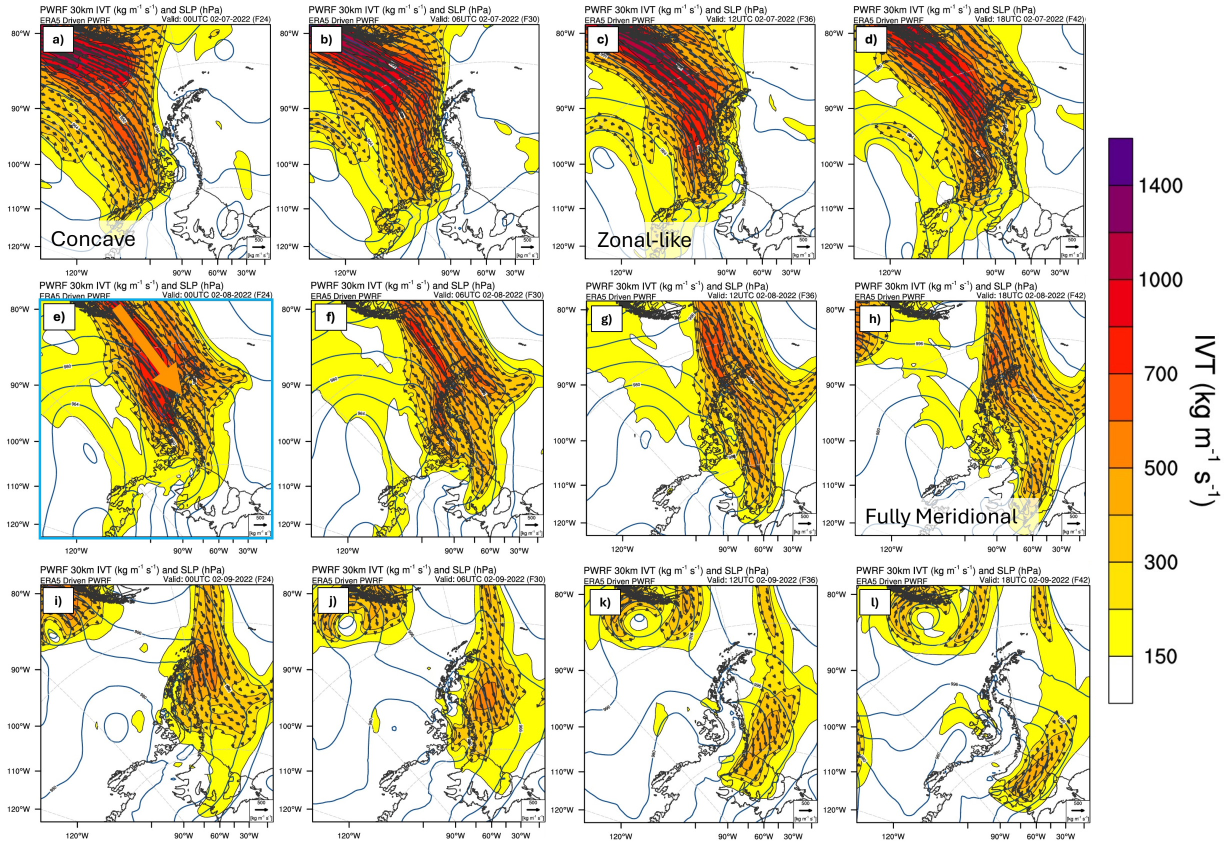

Supplementary Figure S10. Evolution of an atmospheric river (AR) event in 2022. (a) - (l) Six-hourly Integrated Vapor Transport (IVT; contour fill) and mean sea level pressure (MSLP; contour line) from 00Z 7 Feb to 00Z 9 Feb 2022 based on PWRF simulations. (e) highlights the dominant AR configuration for this event, with the blue outline indicating the analysis domain and the orange arrow denoting the dominant AR shape.

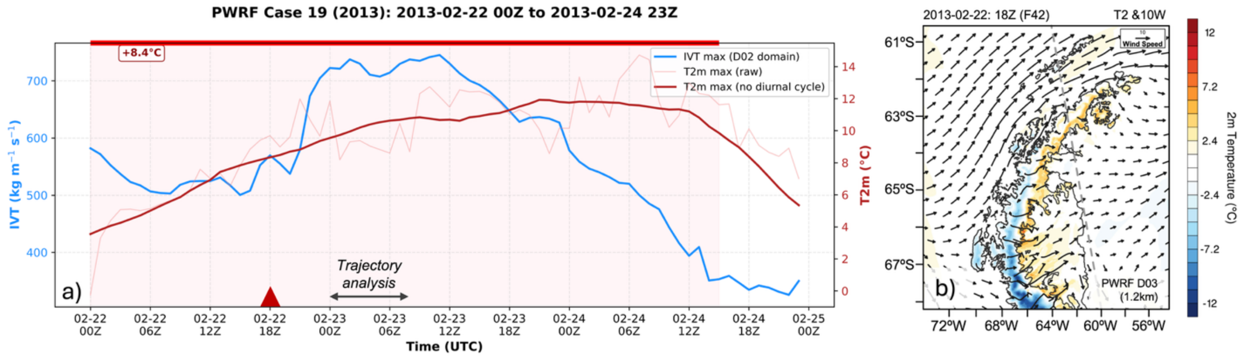

Supplementary Figure S11. Selection of föhn-warming periods for the 2013 convex event. (a) Time series of maximum 2m temperature (light pink line) over the Larsen C Ice Shelf (LCIS), shown with and without removal of the diurnal cycle (solid red line), together with the maximum Integrated Vapor Transport (IVT; blue line) within the PWRF D02 domain. (b) Spatial fields of 2m temperature and 10m wind at 18Z on 22 Feb 2013. In (a), the pink shading denotes the föhn-warming period selected for the k-means clustering analysis. The red triangle at the bottom indicates the time of the snapshot shown in (b), and the black arrowed line marks the time window over which the trajectory analysis was conducted. The length of the reference arrow at the upper right corner in (b) represents a wind speed of 10 m s^{-1}.

References:

1. Zhang, Z. *et al.* Extending the Center for Western Weather and Water Extremes (CW3E) atmospheric river scale to the polar regions. *Cryosphere* **18**, 5239–5258 (2024).
2. Guan, B. [Data] Global Atmospheric Rivers Database, Version 4. UCLA Dataverse, accessed 2025-12-26, https://doi.org/10.25346/S6/ZSW7UN (2025).
3. Zou, X. *et al.* Gridded Polar Atmospheric River (AR) scale dataset. Antarctic Meteorological Research and Data Center, accessed 2024-12-01, https://doi.org/10.48567/ssm5-x097 (2025).
4. Zou, X. *et al.* Selected austral summer Atmospheric River cases over the Antarctic Peninsula, 2001 - 2022. Antarctic Meteorological Research and Data Center, accessed 2024-12-01, https://doi.org/10.48567/exak-2810 (2025).
5. Rowe, P. M., Cordero, R. R., Zou, X. & Gorodetskaya, I. V. Shortwave Downward Radiative Flux (King George Island), 2017 - 2023. Antarctic Meteorological Research and Data Center, accessed 2024-12-01, https://doi.org/10.48567/peeg-7j35 (2025).
6. Rowe, P. M., Cordero, R. R., Zou, X. & Gorodetskaya, I. V. Longwave Downward Radiative Flux (King George Island), 2017 - 2023. Antarctic Meteorological Research and Data Center, accessed 2024-12-01, https://doi.org/10.48567/hnhz-f486 (2025).
7. Gorodetskaya, I. V. *et al.* Record-high Antarctic Peninsula temperatures and surface melt in February 2022: a compound event with an intense atmospheric river. *npj Clim. Atmos. Sci.* **6**, 202 (2023).
8. Ralph, F. M. *et al.* A Scale to Characterize the Strength and Impacts of Atmospheric Rivers. *Bull. Am. Meteorol. Soc.* **100**, 269–289 (2019).

9. Xue, J., Bromwich, D. H., Xiao, Z. & Bai, L. Impacts of initial conditions and model configuration on simulations of polar lows near Svalbard using Polar WRF with 3DVAR. *Q. J. Royal Meteorol. Soc.* **147**, 3806–3834 (2021).
10. Hines, K. M., Bromwich, D. H., Silber, I., Russell, L. M. & Bai, L. Predicting Frigid Mixed-Phase Clouds for Pristine Coastal Antarctica. *J. Geophys. Res. Atmos.* **126**, e2021JD035112 (2021).
11. Hartigan, J. A. & Wong, M. A. A K-Means Clustering Algorithm. *J. R. Stat. Soc. Ser. C. Appl. Stat.* **28**, 100–108 (1979).
12. Zou, X. *et al.* Strong Warming Over the Antarctic Peninsula During Combined Atmospheric River and Foehn Events: Contribution of Shortwave Radiation and Turbulence. *J. Geophys. Res. Atmos.* **128**, e2022JD038138 (2023).
